# Optimum power transfer in RF front end systems using adaptive impedance matching technique

**DOI:** 10.1038/s41598-021-91355-4

**Published:** 2021-06-03

**Authors:** Mohammad Alibakhshikenari, Bal S. Virdee, Leyre Azpilicueta, Chan H. See, Raed Abd-Alhameed, Ayman A. Althuwayb, Francisco Falcone, Isabelle Huynen, Tayeb A. Denidni, Ernesto Limiti

**Affiliations:** 1 Electronic Engineering Department, University of Rome “Tor Vergata”, Via Del Politecnico1, 00133 Rome, Italy; 2 Center for Communications Technology, London Metropolitan University, London, N7 8DB UK; 3 School of Engineering and Sciences, Tecnologico de Monterrey, 64849 Monterrey, NL Mexico; 4 School of Engineering and the Built Environment, Edinburgh Napier University, Edinburgh, EH10 5DT UK; 5 Faculty of Engineering and Informatics, University of Bradford, Bradford, BD7 1DP UK; 6 Department of Electrical Engineering, College of Engineering, Jouf University, Sakaka, 72388 Aljouf Saudi Arabia; 7 Electronic and Communication Engineering Department, Public University of Navarre, 31006 Pamplona, Spain; 8 Institute of Smart Cities, Public University of Navarre, 31006 Pamplona, Spain; 9 Institute of Information and Communication Technologies, Electronics and Applied Mathematics, Université Catholique de Louvain, Ottignies-Louvain-la-Neuve, Belgium; 10 Institut National de la Recherche Scientifique (INRS), Université du Quebec, Montreal, QC H5A 1K6 Canada

**Keywords:** Engineering, Electrical and electronic engineering

## Abstract

Matching the antenna’s impedance to the RF-front-end of a wireless communications system is challenging as the impedance varies with its surround environment. Autonomously matching the antenna to the RF-front-end is therefore essential to optimize power transfer and thereby maintain the antenna’s radiation efficiency. This paper presents a theoretical technique for automatically tuning an *LC* impedance matching network that compensates antenna mismatch presented to the RF-front-end. The proposed technique converges to a matching point without the need of complex mathematical modelling of the system comprising of non-linear control elements. Digital circuitry is used to implement the required matching circuit. Reliable convergence is achieved within the tuning range of the *LC*-network using control-loops that can independently control the *LC* impedance. An algorithm based on the proposed technique was used to verify its effectiveness with various antenna loads. Mismatch error of the technique is less than 0.2%. The technique enables speedy convergence (< 5 µs) and is highly accurate for autonomous adaptive antenna matching networks.

## Introduction

Demand for the higher data rate has necessitated the development new generation of mobile communication systems. Antennas interface the mobile communications devices to the transmission medium, and their performance is affected by the environment including the human body and/or other objects in its proximity. The environment can adversely affect the antenna’s impedance resulting in unwanted mismatch at the input of the RF front-end^1–3^. In the transmission-mode and under the worst-case scenario the mismatch in the impedance can adversely affect the performance of the power-amplifier (PA), which reduces the life of the battery due to excessive energy consumption by the PA^4^. In the receive mode, any mismatch degrades the carrier-to-noise ratio.

To resolve the issue with impedance mismatch, isolators can be used however they can undermine the maximum radiated power and efficiency. In addition, isolators have a narrow bandwidth and therefore are unsuitable for multiband wireless systems. Alternatively, the quality of the link can be maintained by applying adaptive impedance matching techniques^5,6^. This technique is popular for maintaining system performance parameters such as optimum radiated power, linearity of PA, sensitivity of receiver, and power-efficiency. Moreover, its applicable in wireless systems operating at multiple bands as it enables a single impedance matching network (IMN) to suffice. However, the use of adaptive IMN in wireless systems are incumbered by stringent criteria on insertion-loss (*IL*), degree of linearity, and tuning span. The use of adaptively controlled IMNs^7,8^ is only possible with the availability of highly linear and high Quality-factor tuneable components such as RF microelectromechanical (MEM) devices^9,10^, CMOS-switches^11,12^, silicon and Barium-Strontium-Titanate (BST) varactor diodes^13,14^.

Recent works reported in literature on adaptive impedance-matching include: (i) a T-shaped adaptive impedance matching system that refers to predetermined load-Q information for different matching conditions to implement the impedance matching^15^. The T-shaped network uses tuneable capacitors that are controlled by digital relays. The frequency range for tuning is limited to between 10 and 95 MHz; (ii) the use of fuzzy inference system to construct the mapping relationship between load impedance and the matched capacitor set^16^. This technique is applied to optimise power transfer between coupled coils at a fixed frequency; (iii) the use of a machine learning strategy based on neural networks for the real-time range-adaptive automatic impedance matching of wireless power transfer applications^17^. Here the voltage controlled variable capacitors are employed in a π-type matching circuit. The matching is implemented for different gap spacing between the transmitter and receiver coils at a fixed frequency; and (iv) using RF MEMS based on a coplanar waveguide based on suspended bridges for impedance tuning^18^. The tuning is controlled by a variable applied DC voltage to the bridges over 1–6 GHz.

This paper presents the theory for an effective adaptive antenna impedance matching technique. The IMN includes control-loops to independently control the impedance. In fact, it uses sensors to monitor the voltage/current fluctuations in the matching network to reliably control the real and imaginary parts of impedance and thereby reduce *IL*. The proposed technique is designed to operate autonomously to provide conjugate matching over a finite frequency range at which the system operates, and its effectiveness was verified using an algorithm based on the technique. It should be noted that in transmit mode the load impedance of a transceiver can be affected by the nonlinearity effect of the power amplifier when it is operated in saturation mode. Nowadays, the effects of non-linearity are negated by using digital predistortion^19^. Alternatively, load impedance mismatch can be avoided by using an isolator at the amplifier output. It is assumed here that the effects of harmonics are mitigated using one of these techniques.

Rest of the paper is organized as follow as. The proposed approach to control antenna impedance match is described in the next section. Then, controlling the matching impedance of the antenna based on *LC-*network is demonstrated. After that, the LC-network adjusting zone is presented. Then, daptive control of parallel *LC*-network is discussed. Afterward, he proposed technique is compared with state-of-the-art IMNs. Finally, the paper is concluded.

## Proposed approach to control antenna impedance matching network

**Figure 1:**
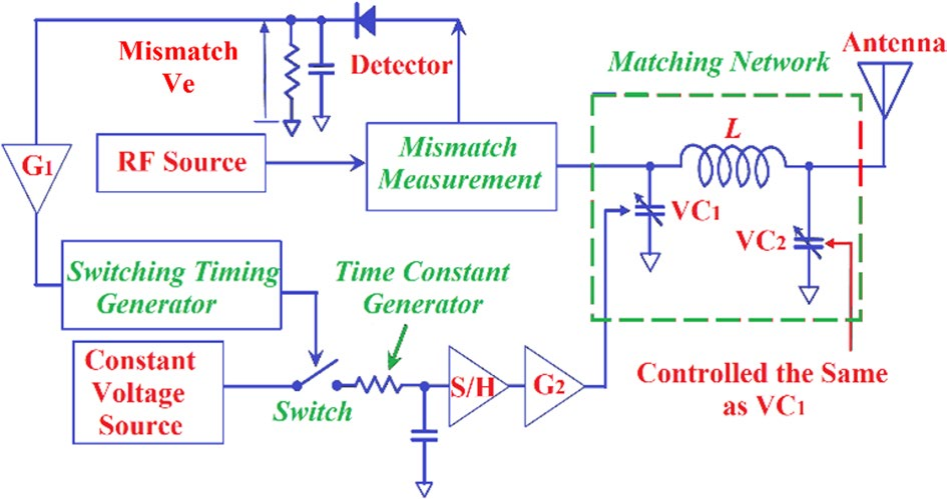
Configuration of the proposed adaptive matching system.

The configuration of the proposed adaptive matching system is shown in Fig. 1. In the transmit mode it consists of a matching network, directional coupler for mismatch measurement, a switch, switch timing generator, and time constant generator. Varactor diodes in the matching network provide electronically controllable capacitance. The system uses the magnitude of the return-loss (*Г*) that is measured between the RF-source and the matching circuit. As information on the phase of *Г* is also essential to minimize the degree of mismatch, the system uses a test signal to determine whether the mismatch increases or decreases. This information is used to precisely control the capacitance in the matching network.

Protocol used here for adaptively matching the impedance involves measuring the degree of mismatch with the detection circuitry. This is achieved by turning the switch ‘on’ to increase the control voltage to the varactor#1 (VC_1_). If the mismatch worsens the system acknowledges this and turns the switch ‘off’. If the mismatch reduces the system acknowledges this by keeping the switch ‘on’. This is maintained for the period of the control frame for VC_1_. In the time frame period of VC_1_, the control voltage to the varactor#2 (VC_2_) is maintained at the value of the last time frame of the VC_2_. The voltage is maintained using the sample-and-hold circuitry. At the end of the time frame period of VC_1_, the control voltage for VC_1_ is maintained and the time frame period commences for VC_2_. Compared to other conventional techniques that use the steepest descent algorithm for optimization, the merits of the proposed system are: (i) no need for complex mathematical modelling; (ii) the nonlinearity of the control elements (varactor-diode) are considered to realise rapid convergence of impedance matching; and (iii) varactor-diodes of any range of capacitance are applicable. As it is not possible to obtain a desired varactor-diode with the required capacitance range the only option therefore is to use an available varactor-diode with a broader capacitance range. In the system an appropriate inductance *L* needs to be chosen, which is determined by simulation through parametric analysis. To characterise the improvement in impedance mismatch, we used time characteristics of the return-loss between the matching network and the RF-front-end.

## Controlling antenna-impedance matching based on *LC*-network

**Figure 2:**
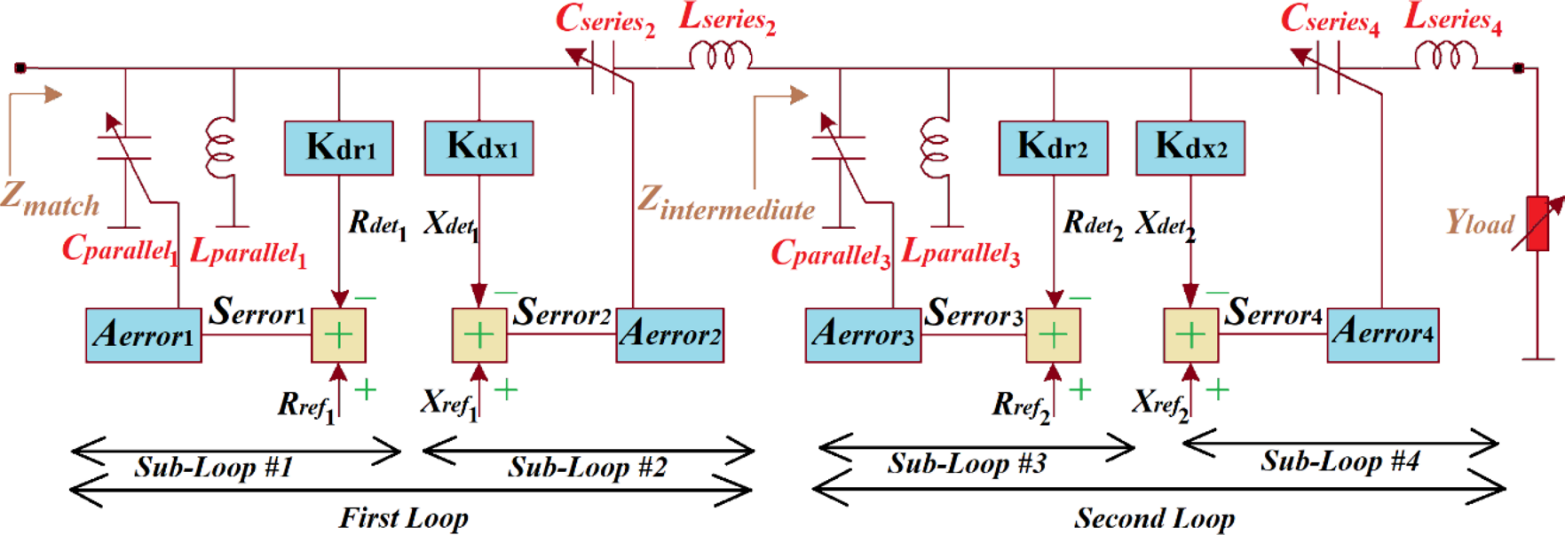
Schematic diagram for independent control of the matched impedance ${Z}_{match}$ of an *LC-*network.

In the proposed technique the *LC-*network is extended in comparison to^2^ to include two loops comprising a serial *LC* sub-loop and a parallel *LC* sub-loop that are independent from each other, as shown in Fig. 2. These loops now control components constituting the impedance matching network. The control loops essentially convert an undefined load admittance *Y*_*load*_ to the required matching impedance ${Z}_{match}$ represented by^2^:

1 $${{{Z}_{match}=R}_{match}+jX}_{match}$$

The loop#1 controls the parallel and series capacitors ${C}_{{parallel}_{1}}$ and ${C}_{{series}_{2}}$, respectively, representing the imaginary-part of the match impedance ${X}_{match}$. The loop#2 controls the parallel and series capacitors ${C}_{{parallel}_{3}}$ and ${C}_{{series}_{4}}$, respectively, to set the real-part of the match impedance ${R}_{match}$. The intermediate impedance (${Z}_{intermediate}$) is given by:

2 $${{{Z}_{intermediate}=R}_{intermediate}+jX}_{intermediate}$$

If loop#2 is frozen and the amplifier-gain errors ${A}_{{error}_{3}}$ and ${A}_{{error}_{4}}$ become significant, the signal errors ${S}_{{error}_{3}}$ and ${S}_{{error}_{4}}$ will too be insignificant.

3 $${R}_{intermediate}=1+\frac{{R}_{ref2}}{{K}_{dr2}}$$

where ${R}_{ref2}$ and ${K}_{dr2}$ are the magnitude of the reference and the detector constant, respectively, of loop#2 that sets the magnitude of ${R}_{match}$. Loop#2 introduces an intermediate reactance defined by:

4 $${X}_{intermediate}=\frac{{K}_{dx2}}{{X}_{ref2}}-1$$

where ${X}_{ref2}$ and ${K}_{dx2}$ are the magnitude of the reference and the detector constant, respectively, of loop#2 setting the magnitude of ${X}_{match}$. Similarly, if loop#1 is frozen and the amplifier-gain errors ${A}_{{error}_{1}}$ and ${A}_{{error}_{2}}$ are significant, the signal errors ${S}_{{error}_{1}}$ and ${S}_{{error}_{2}}$ will be insignificant and, by approximation, hold true:

5 $${R}_{match}=2+\frac{{R}_{ref1}}{{K}_{dr1}}+\frac{{R}_{ref2}}{{K}_{dr2}}$$

where ${R}_{ref1}$ and ${K}_{dr1}$ are the magnitude of the reference and the detector constant, respectively, of loop#1 that sets the magnitude of ${R}_{match}$. Loop#1 introduces an intermediate reactance ${X}_{match}$ defined by:

6 $${X}_{match}=\frac{{K}_{dx1}}{{X}_{ref1}}+\frac{{K}_{dx2}}{{X}_{ref2}}$$

where ${X}_{ref1}$ and ${K}_{dx1}$ are the reference value and the detector constant, respectively, of the first loop that sets the magnitude of ${X}_{match}$. From Eq. (1) ${Z}_{match}$ can be written as

7 $${Z}_{match} =2+\frac{{R}_{ref1}}{{K}_{dr1}}+\frac{{R}_{ref2}}{{K}_{dr2}}+j \left(\frac{{K}_{dx1}}{{X}_{ref1}}+\frac{{K}_{dx2}}{{X}_{ref2}}\right)$$

Equation (7) shows that the matched impedance is not dependent on ${Y}_{load}$, the amplifier gain errors, and the magnitude of the matching-network components.

**Figure 3:**
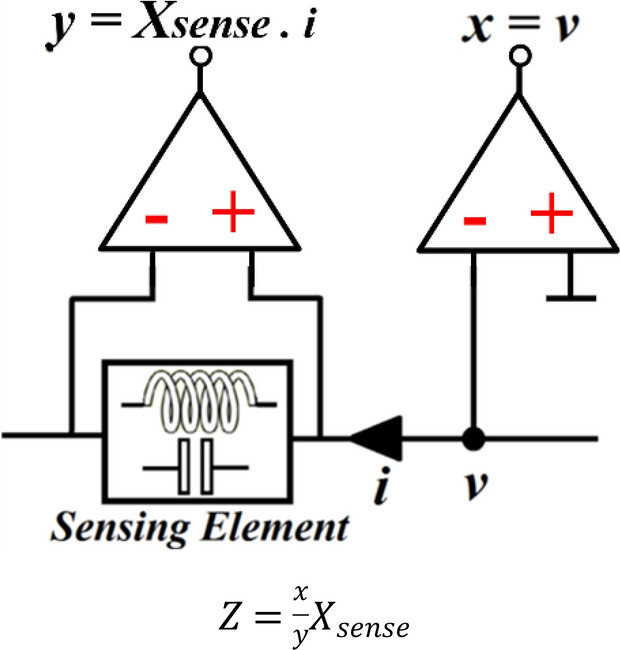
Impedance (*Z*) can be deduced by sensing the voltage “*v*” and current “*i*”.

Monitoring impedance mismatch involves monitoring of RF signal and converting it to *dc*. As the impedance is a function of voltage and current, the RF voltage or its corresponding current can be ‘sensed’ to establish the impedance. Figure 3 shows the point of voltage measurement *“v”*. The differential voltage across a monitoring component is used to measure the current *“i”* and hence its reactance ${X}_{sense}$ can be determined. The monitoring component can be either an inductance or capacitance that is part of the tuneable IMN. The impedance (*Z*) is determined by taking the ratio of the two buffer-amplifier outputs in Fig. 3.

**Figure 4:**
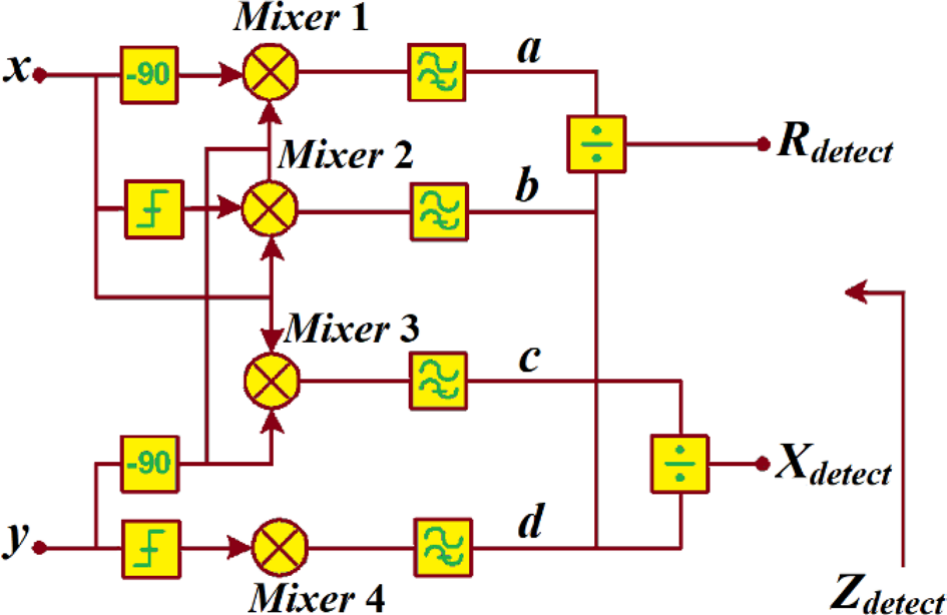
Quadrature detector to generate the constituent parts of the detected impedance from the return-loss.

Individual components representing the impedance can be determined by applying the *RF* signal’s magnitude ‘*x’* and *‘y’* in Fig. 3 into the detector configuration in Fig. 4, where *‘x’* can be represented by:

8 $$x={A}_{x}\mathrm{cos}(\omega \cdot t+{\theta }_{x})$$

and current information to input *“y”* is represented by:

9 $$y={A}_{y}{\cos}\left(\omega \cdot t+{\theta }_{y}\right) \cdot \left|\frac{1}{{X}_{sense}}\right| \cdot {e}^{j\frac{\pi }{4}}$$

Input signal *‘x’* is fed to *mixer*#1 with 90° phase shift, to *mixer*#2 with a limited amplitude, and to *mixer*#3. Whereas input signal *‘y’* is fed to the same mixers with 90° phase shift as well as *mixer*#4 with limited amplitude. This configuration generates phase differential between *‘x’* and *‘y’* corresponding to magnitudes ${A}_{x}$ and ${A}_{y}$, respectively. *Mixers*#1 & #2, and *mixers#*3 & #4 are also used to find the magnitudes of ${A}_{x}$ and ${A}_{y}$ of input signals *‘x’* and *‘y’*, respectively. The output signal of *mixers*#1 & #2 is split by *mixers*#3 & #4 and vice versa to obtain the detected impedance ${Z}_{detect}$, represented by:

10 $${{Z}_{detect}=R}_{detect}+j{X}_{detect}$$

where $${R}_{detect}=a \cdot b/d$$ and $${X}_{detect}=c \cdot d/a$$.

11 $${\theta }_{detect}={\theta }_{x}-{\theta }_{y}$$

where *a*, *b*, *c*, and *d* are defined as:

12 $$a=2\pi {A}_{x}\mathrm{cos}\left({\theta }_{detect}\right) \cdot \frac{1}{{X}_{sense}}$$

13 $$b=\frac{\pi }{2}{A}_{x}\mathrm{sin}\left({\theta }_{detect}\right)$$

14 $$c=\pi {A}_{y}\mathrm{cos}\left({\theta }_{detect}\right)$$

15 $$d=\frac{\pi }{2}{A}_{y}\mathrm{sin}\left({\theta }_{detect}\right) \cdot \frac{1}{{X}_{sense}}$$

From Eqs. (12)–(15), the real and imaginary parts of the detected impedance are given by:

16 $${R}_{detect}=\frac{2\pi {A}_{x}^{2}\mathrm{cos}({\theta }_{detect})\mathrm{sin}({\theta }_{detect}) }{{A}_{y}\mathrm{sin}\left({\theta }_{detect}\right)}$$

17 $${X}_{detect}=\frac{\pi {A}_{y}^{2}\mathrm{cos}\left({\theta }_{detect}\right)\mathrm{sin}\left({\theta }_{detect}\right)}{4{A}_{x}\mathrm{cos}({\theta }_{detect})}$$

By combining Eqs. (16) and (17) the impedance detected is given by:

18 $${Z}_{detect}=\frac{2\pi {A}_{x}^{2}\mathrm{cos}({\theta }_{detect})\mathrm{sin}({\theta }_{detect}) }{{A}_{y}\mathrm{sin}\left({\theta }_{detect}\right)}+j\frac{\pi {A}_{y}^{2}\mathrm{cos}\left({\theta }_{detect}\right)\mathrm{sin}\left({\theta }_{detect}\right)}{4{A}_{x}\mathrm{cos}({\theta }_{detect})}$$

According to Eqs. (16) and (17), the detected values of the impedance are independent of the frequency. This means frequency compensation is not required for high accuracy across a wideband frequency. The detected values of the impedance are related to the ratios $\frac{{A}_{x}}{{A}_{y}}$ and $\frac{{A}_{y}}{{A}_{x}}$, hence they are independent of *RF* signal power transmitted. Moreover, according to Eq. (11), ${\theta }_{detect}$ is the differential phase between ${\theta }_{x}$ and ${\theta }_{y}$.

By simply exchanging the detector input signals *‘x’* and *‘y’* the detector generates the real and imaginary parts of the admittance. When input signals *‘x’* and *‘y’* are the reflected and incident power, the detector generates a reading representing the return-loss (*Г*). The accuracy of the detector is essentially dependent on the limiter and its amplitude dependent phase-delay. The detector needs to be operated at a lower ‘on/off’ duty-cycle (< 1%) to conserve power since the settling time of the detector is normally short (10–100 µs) compared to the impedance variation of the antenna. The detector is susceptible to receiving unwanted signals as it is not frequency selective device. The signals *‘x’* and *‘y’* can cause the direction of the current flow to change when they are stronger than the transmit signal. In that case the detector reads the network impedance seen in the reverse direction. However, at lower output power (< 0 dBm), there is no advantage from adaptive impedance matching. In this case the detector can be turned ‘off’ to prevent erroneous control.

### Series LC-network

**Figure 5:**
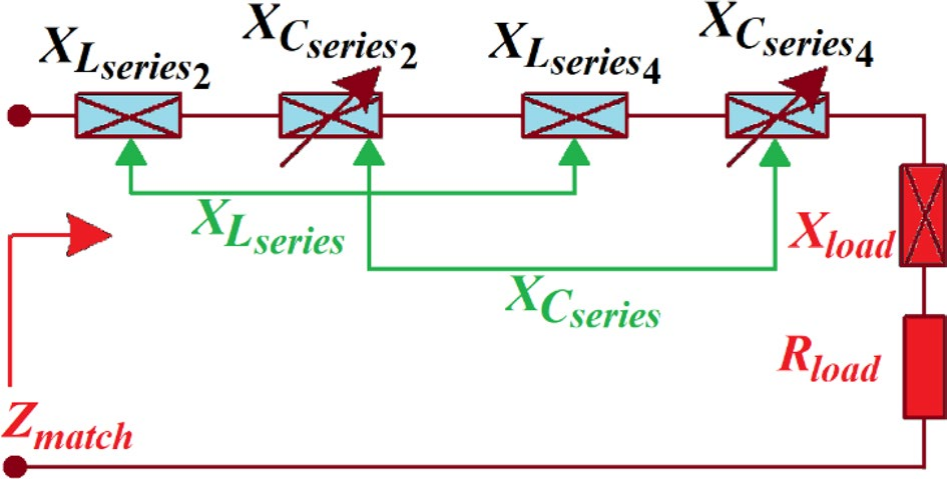
Adjustable *LC*-network to provide the required inductive and capacitive load reactance.

In Fig. 5, the matched impedance ${Z}_{match}$ of a series *LC*-network represents the tuneable network of the sub-loops 3 and 4 of the first and second loops, respectively, in Fig. 2, and is given by:

19 $${Z}_{match}={R}_{match}+j{X}_{match}$$

where $${X}_{match}={X}_{{L}_{series}}+{X}_{{C}_{series}}+{X}_{load}$$, $${X}_{{L}_{series}}={X}_{{L}_{series 2}}+{X}_{{L}_{series 4}}$$, $${X}_{{C}_{series}}={X}_{{C}_{series 2}}+{X}_{{C}_{series 4}}$$ and $${R}_{match}={R}_{load}$$.

Tuning the series capacitor values $${C}_{series}$$ affects $${X}_{match}$$, which is a function of tuning reactance ($${X}_{{C}_{series}})$$, whereas the matched series resistance ($${R}_{match}$$) is equivalent to load resistance ($${R}_{load}$$). In adaptive matching networks, the orthogonal property of resistance and reactance is exploited in the adaptive *LC*-network to modify the matched reactance $$({X}_{match}$$) to the required value without affecting the matched resistance $$({R}_{match}$$).

The proposed series *LC*-network is used to alter the real-part of the matched admittance. The matched impedance ($${Z}_{match})$$ can be represented by matched admittance given by:

20 $${Y}_{match}={G}_{match}+j{B}_{match}$$

where $${G}_{match}=\frac{{({R}_{load})}^{2}+{({X}_{match})}^{2}}{{R}_{load}}$$ and $${B}_{match}=\frac{{({R}_{load})}^{2}+{({X}_{match})}^{2}}{{X}_{match}}$$.

**Figure 6:**
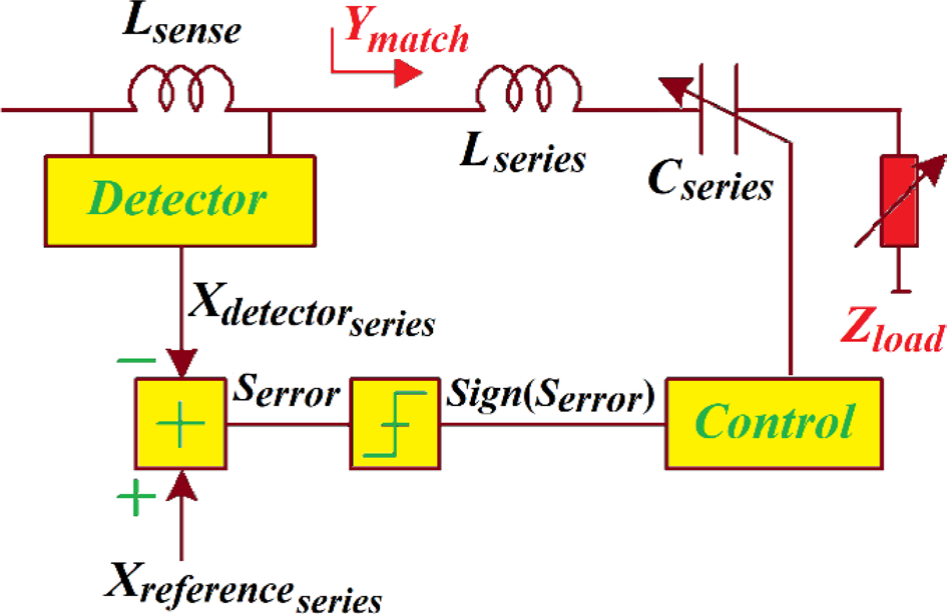
Series *LC*-network to control the real-part of the matched admittance ${G}_{match}$.

The matched conductance $({G}_{match})$ is a symmetric function of ${X}_{match}$. Consequently, a series *LC*-network, shown in Fig. 6, can convert load resistance ${R}_{load}$ to a conductance that is smaller than $1/{R}_{load}$. Two solutions exist for the condition ${G}_{match}<1/{R}_{load}$ given by:

21 $${X}_{match}=\frac{{R}_{load}}{{G}_{match}}\sqrt{(1+{G}_{match}{R}_{load})}$$

Substitution of Eq. (21) into (20) gives corresponding matched susceptance given by

22 $${B}_{match}=\sqrt{\left(\frac{{R}_{load}}{{G}_{match}}+{G}_{match}{R}_{load}\right)+1}$$

### Parallel LC-network

**Figure 7:**
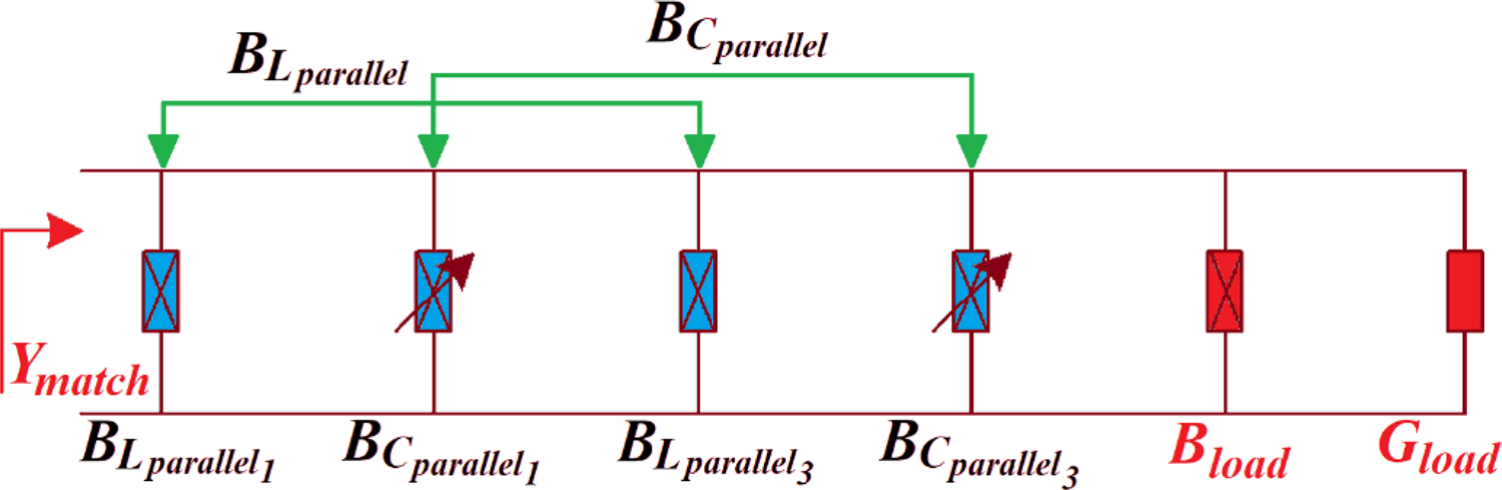
Variable parallel *LC*-network and its matched admittance ${Y}_{match}$.

Figure 7 shows the matched admittance ${Y}_{match}$ of a parallel *LC* network and representing the tuneable network of the sub-loops 1 and 3 of the first and second loops, respectively, (see Fig. 2), is defined as

23 $${Y}_{match}={G}_{match}+j{B}_{match}$$

where $${B}_{match}={B}_{{L}_{parallel}}+{B}_{{C}_{parallel}}+{B}_{load}$$, $${B}_{{L}_{parallel}}={B}_{{L}_{parallel} 1}+{B}_{{L}_{parallel} 3}$$, $${B}_{{C}_{parallel}}={B}_{{C}_{parallel} 1}+{B}_{{C}_{parallel} 3}$$ and $${G}_{match}$$ = $${G}_{load}$$.

Matching admittance ($${Y}_{match})$$ of this parallel *LC*-network corresponds to $${Y}_{intermediate}$$ of the *LC-*network. The matched susceptance $$({B}_{match})$$ is a function of tunable susceptance ($${B}_{{C}_{parallel})}$$, whereas the matched conductance $${G}_{match}$$ is equal to load conductance $${G}_{load}$$ and independent $${B}_{{C}_{parallel}}$$. $${Y}_{match}$$ and the orthogonal property of conductance/susceptance can be exploited for adaptive control of the IMN by tuning the matched susceptance to the required value without adversely affecting the matched conductance.

Moreover, the parallel *LC*-network can be used to control the real-part of $${R}_{match}$$. Matched impedance ($${Z}_{match}=1/{Y}_{match})$$ is represented by:

24 $${Z}_{match}={R}_{match}+j{X}_{match}$$

where $${R}_{match}=\frac{{G}_{load}+{B}_{match}}{{({G}_{load})}^{2}}$$ and $${X}_{match}=\frac{{G}_{load}+{B}_{match}}{{({B}_{match})}^{2}}$$.

**Figure 8:**
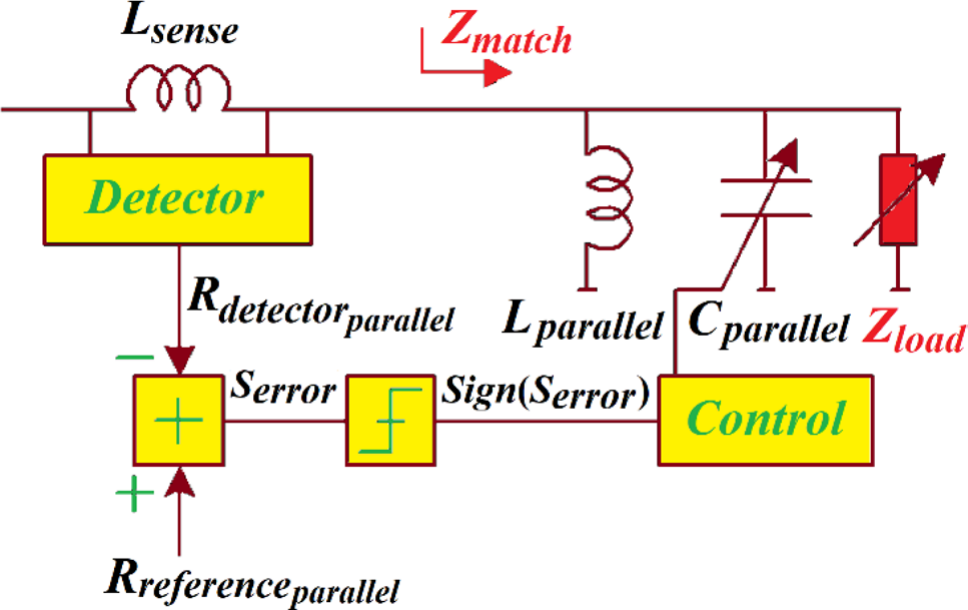
Parallel *LC*-network to control the real-part of the matched impedance ${R}_{match}$.

In the parallel *LC*-network to control the real-part of the matched impedance in Fig. 8, ${R}_{match}$ is a symmetric function of ${B}_{match}$.

Hence, a parallel *LC*-network transforms load conductance $({G}_{load})$ to a resistance that is smaller than $1/{R}_{load}$. When ${R}_{match}<1/{G}_{load}$, the solution obtained from Eq. (24) is given by:

25 $${B}_{match}=\frac{{R}_{match}}{{G}_{load}}\sqrt{(1+{R}_{match}{G}_{load})}$$

## *LC*-network adjusting zone

The *LC*-network impedance adjusting zone is determined by the relationship between the impedance correction and the required capacitor’s adjusting range for the *LC-*network. As the impedance transformation needs to be done in two steps, the intermediate impedance ${Z}_{intermediate}$ is first defined. This impedance is used to transform an arbitrary load-admittance ${Y}_{load}={G}_{load}+j{B}_{load}$ to the required ${Z}_{match}={R}_{match}+j{X}_{match}$. For this reason, the parallel section converts the ${Y}_{load}$ to a transitional impedance, whose real-part ${R}_{intermediate}$ should be equal to the ${R}_{match}$. The real-part of intermediate impedance can be shown to be given by:

26 $${R}_{intermediate}={R}_{match}=\frac{{({G}_{load})}^{2}+{({B}_{load}+{B}_{series}+{B}_{parallel})}^{2}}{{G}_{load}}$$

and the imaginary-part of this intermediate impedance is given by:

27 $${X}_{intermediate}={R}_{match}\sqrt{\frac{{R}_{match}{+G}_{load}}{{R}_{match}{G}_{load}+1}}$$

and the corresponding intermediate susceptance is given by:

28 $${B}_{intermediate}={G}_{load}\sqrt{\frac{{R}_{match}{+G}_{load}}{{R}_{match}{G}_{load}+1}}$$

The required parallel capacitor ${C}_{parallel}$ is given by:

29 $${C}_{parallel}=\frac{4\pi f}{2\pi f({B}_{intermediate}-{B}_{load}+{B}_{{L}_{parallel}})}$$

Equations (26) and (27) define ${C}_{parallel}$ that is needed to realise a desired correction from a load ${G}_{load}+j{B}_{load}$ to a matched resistance ${R}_{match}$ at frequency *f* and parallel inductor susceptance ${B}_{{L}_{parallel}}$. The required magnitude of capacitance can be obtained from:

30 $${C}_{series}=2\pi f({X}_{intermediate}+{X}_{match}+{X}_{{L}_{series}})$$

**Figure 9:**
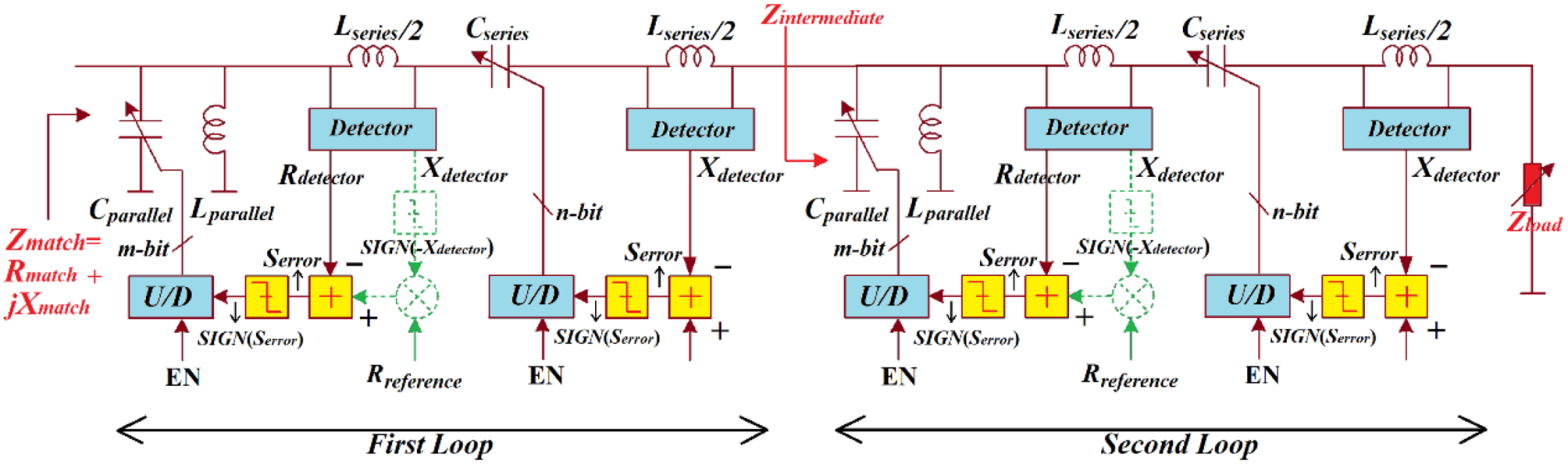
*LC*-network implemented. An extra feedback path is shown in dotted ‘green’ blocks. This feedback ensures the first and second loops to function in their stable regions.

Equations (26) and (27) define the series capacitance ${C}_{series}$ that is required to correct from a load ${G}_{load}+j{B}_{load}$ to a matched resistance ${R}_{match}$ and a matched reactance ${X}_{match}$ at a frequency *f* and known inductor reactance ${X}_{{L}_{series}}$. It should be noted that ${C}_{series}$ is independent of load susceptance. Furthermore, to realise a physically realizable solution, these two formulations are valid when the series and parallel capacitors and the square-root argument are positive. The last condition is met when ${R}_{match}<1/{G}_{load}$. For real-to-real impedance conversion, this transformation can only be descending, which outlines the impedance adjusting zone. The boundary condition for the up-converting *LC*-network in Fig. 9 is obtained when

31 $${B}_{{L}_{parallel}}<{B}_{intermediate}+{B}_{load}$$

32 $$\text{and} \;\;{X}_{{L}_{series}}<{X}_{intermediate}-{X}_{match}$$

The impedance adjusting range is bounded by fixed inductors ${L}_{parallel}$ and ${L}_{series}$. Susceptance of the parallel inductor sets the correction limit of the capacitive mismatch, and the reactance of the series inductor sets the correction limit of the capacitive intermediate impedances.

Capacitance ratios to transform an arbitrary load admittance ${Y}_{load}={G}_{load}+j{B}_{load}$ to a required matched impedance ${Z}_{match}$ need to be determined. To do this the network should be able to adjust load admittance ${Y}_{load1}={G}_{load1}+j{B}_{load1}$ to match with impedance ${Z}_{match1}$ with a real-part ${R}_{match1}$, at a given frequency *f*_*1*_, by a parallel capacitance ${C}_{parallel1}$ and a series capacitance ${C}_{series1}$. Furthermore, the same network must be able to tune a load admittance. ${Y}_{load2}={G}_{load2}+j{B}_{load2}$ to ${Z}_{match2}$ with resistance. ${R}_{match2}$, at a frequency *f*_*2*_, by capacitance ${C}_{parallel2}$ and a series capacitance ${C}_{series2}$. From Eq. (27), the required capacitance adjusting zone of ${C}_{{Z}_{{C}_{parallel}}}$, given by the capacitor ratio, can be expressed as:

33 $${C}_{{Z}_{{C}_{parallel}}}=\frac{{f}_{1}}{{f}_{2}}\left\{\frac{{B}_{intermediate2}+{B}_{load2}+{B}_{{L}_{parallel2}}}{{B}_{intermediate1}-{B}_{load1}-{B}_{{L}_{parallel1}}}\right\}$$

Similarly, from Eq. (30), the series capacitor is given by:

34 $${C}_{{Z}_{{C}_{series}}}=\frac{{f}_{1}}{{f}_{2}}\left\{\frac{{X}_{intermediate1}+{X}_{match1}+{X}_{{L}_{series1}}}{{X}_{intermediate2}-{X}_{match2}-{X}_{{L}_{series2}}}\right\}$$

The above equations yield four solutions at the two frequencies (*f*_*1*_ and *f*_*2*_). This is because we can realise matching by transforming the inductive or capacitive intermediate impedance. In addition, the equations reveal the capacitance ratio is proportional to the frequency range of operation.

## Adaptive control of parallel *LC*-network

An algorithm was developed to determine the convergence operation of the control loop. In fact, the tuneable capacitor, which is essentially a switched-capacitor array, is controlled by the sign of the error signal *Sign*(${S}_{error}$) generated from the series and parallel control loops. The up/down counter (U/D) is used to store the value of the control array. The output of the U/D is incremented or decremented in steps of one least significant bit, which depends on the error signal.

The convergence operation can be examined in open-loops conditions. At the controller when the loops (Figs. 6 and 8) are opened we ‘sense’ *Sign*(${S}_{error}$) across the entire ranges of ${X}_{match}$ and ${B}_{match}$, *Sign*(${S}_{error}$) is + 1. Hence, the directions of capacitor controls shown in Figs. 6 and 8 are not definitive. This can be resolved by using the detected information on the signs of the matched susceptance *Sign*(*-*${B}_{match}$) and the matched reactance *Sign*(*-*${X}_{match}$), respectively, as a secondary control criterion shown in Fig. 9 by dotted green blocks. The secondary feedback path allows the series and parallel control loops criteria to be determined by the two detection thresholds for each loops of ${G}_{match}$ = ${R}_{{reference}_{series}}$ and ${B}_{match}$ = 0 (for series control loop shown in Fig. 6) and ${R}_{match}$ = ${R}_{{reference}_{parallel}}$ and ${X}_{match}$ = 0 (for parallel control loop shown in Fig. 8). Assuming that detector constants ${K}_{series}$ and ${K}_{parallel}$ are equated to unity, ${S}_{error}$ can be represented by:

35 $${S}_{{error}_{series}}=Sign\left(-{B}_{match}\right) \cdot {R}_{{reference}_{series}}{-R}_{{detector}_{series}}$$

36 $${S}_{{error}_{parallel}}=Sign\left(-{X}_{match}\right) \cdot {R}_{{reference}_{parallel}}{-R}_{{detector}_{parallel}}$$

As ${R}_{reference}$, ${R}_{match}$ and ${G}_{match}$ are always positive, error signal ${S}_{error}$ becomes strongly negative when *Sign*(*-*${X}_{match}$) and *Sign*(*-*${B}_{match}$) are negative.

The impedance matching network characteristics are applied to create an adaptive *LC*-network that comprises a pair of loops, as shown in Fig. 9. Control loop#1 sets the real-part of ${R}_{match}$. The sensing inductor ${L}_{sense}$ consists of the series *LC*-network controlled by both loops. The loop#2 transforms the matched reactance ${X}_{match}$ to ${X}_{reference}$. In fact, no information on the real-part of the matched impedance is required. If ${X}_{match}$ is controlled iteratively the sign of ${X}_{detector}$ is significant. The first and second loops converge reliably when operating over the entire range of ${B}_{match}$ and ${X}_{match}$.

An algorithm was created to verify the proposed technique using an arbitrary fixed series and parallel capacitance range. Signal delay in the various circuit components were taken into account in the algorithm. Table 1 shows the target antenna impedance (real and imaginary) required to match with a 50 Ω RF-front-end of a wireless transceiver. It also shows the actual values achieved and the elapsed time to reach the required load impedance. A wide selection of target impedances was used in the simulation to demonstrate its effectiveness. The maximum mismatch error in achieving the desired antenna impedance is 0.17% and the elapsed time to reach the required target impedance is under 5 microseconds. The proposed technique is significantly faster than the authors’ previous technique based on quantum inspired genetic optimization technique^20^, which was experimentally verified.

**Table 1:** Simulation results of target antenna impedance achieved.

Target antenna impedance (Ω)		Matched impedance achieved (Ω)		Mismatch error (%)		Elapsed convergence time (µs)
Real	Imaginary	Real	Imaginary	Real	Imaginary	
100	+ 50	99.94	+ 49.98	0.06	0.02	4.71
100	− 50	99.93	− 49.99	0.07	0.01	4.72
75	+ 85	74.87	+ 84.97	0.13	0.03	4.86
75	− 85	74.89	− 84.87	0.11	0.13	4.91
30	+ 50	29.95	+ 49.96	0.05	0.04	4.67
30	− 50	29.96	− 49.94	0.04	0.06	4.69

## State-of-the-art IMNs comparison

In this section, the characteristics of the proposed impedance matching network is compared with other recent state-of-the-art techniques reported to date. The comparison of salient parameters is shown in Table 2. The convergence time to reach the desired antenna impedance of the proposed technique is comparable to^21^ however not reported is mismatch error. The novelty of the propose work are: (i) automated tuning of *LC* impedance matching network to compensate for antenna mismatch with the RF-front-end; (ii) use of a tuning algorithm that converges to a matching point without the need of complex mathematical modeling of the system and nonlinear control components (varactor-diode), which leads to a rapid convergence; (iii) commercial varactor-diodes with any given capacitance range are applicable; (iv) rapid control is achieved with digital circuitry; (v) reliable convergence is realized inside the tuning range of the *LC*-network; (vi) reduces insertion-loss by using matching network elements to monitor voltage/current signals; and, (vii) enables autonomous control of adaptive antenna matching networks for optimum power transfer.

**Table 2:** State-of-the-art IMNS comparison.

Refs.	Methodology	Impedance Type	Structure	Implementation	Bandwidth	Convergence time	Notes
^2^	Numerical	Insertion loss	Flexible	Combination	Narrow	0.15 s	Complex control system, automated tuning, simple analogue components, complex mathematical modeling
^4^	Analytical	Single	Flexible	Combination	Wide	4.25 s	alleviated method, linear control components, simple analogue components, automated tuning
^15^	Numerical	Single	Fixed	Passive	Narrow	NR	Complex mathematical modeling, alleviated method, complex control system
^16^	Numerical	Single	Fixed	Passive	Narrow	4 s	Transformers used, complex control system, expensive transformers, nonlinear control components
^17^	Analytical	Insertion loss	Fixed	Combination	Narrow	NR	Complex control system, nonlinear control components, transformers used
^18^	Analytical	Insertion loss	Fixed	Passive		NR	Alleviated method, complex mathematical modeling, heavy and expensive transformers
^20^	Numerical	Insertion loss	Flexible	Combination	Wide	14 s	Automated tuning based on quantum inspired genetic optimization technique
^21^	Analytical	Single	Flexible	Combination	Narrow/Wide	4 µs	Transformers used, Complex control system, nonlinear control components
^22^	Analytical	Single	Fixed	Combination	Narrow	NR	Transformers used, alleviated method
^23^	Analytical	Insertion loss	Fixed	Passive	Narrow	NR	Bulky, heavy and expensive transformers
^24^	Numerical	Single	Flexible	Passive	Wide	NR	Complex control system, nonlinear control components
^25^	Analytical	Single	Flexible	Combination	Narrow	NR	Control system not discussed
^26^	Analytical	Single	Fixed	Combination	Narrow	NR	Complex control system, alleviated method
^27^	Analytical	Insertion loss	Fixed	Passive	Narrow	NR	Bulky, heavy and expensive transformers
^28^	Numerical	Insertion loss	Fixed	Passive	Narrow	NR	Transformers used, complex control system, expensive transformers, nonlinear control components
^29^	Analytical	Insertion loss	Fixed	Passive	Narrow	NR	Alleviated method, complex control system, expensive transformers
^30^	Analytical	Insertion loss	Fixed	Combination	Narrow	NR	Transformers used, bulky, complex control system, expensive transformers
^31^	Numerical	Insertion loss	Fixed	Combination	Narrow	NR	Alleviated method, complex mathematical modeling, expensive transformers
^32^	Numerical	Insertion loss	Flexible	Passive	Narrow	NR	Complex control system, nonlinear control components, transformers used
^33^	Analytical	Insertion loss	Fixed	Passive	Wide	NR	Alleviated method, transformers used, nonlinear control components, complex control system
^34^	Analytical	Single	Fixed	Combination	Wide	NR	Fully integrated, tuning for load (antenna) matching, nonlinear control components
^35^	Analytical	Insertion loss	Fixed	Passive	Narrow	NR	Alleviated method, complex control system, fast operating speed, low development cost
^36^	Analytical	Insertion loss	Fixed	Passive	Narrow	NR	Transformers used, alleviated method, complex control system, nonlinear control components
^37^	Analytical	Insertion loss	Flexible	Combination	Narrow	NR	Fully integrated, complex control system, expensive transformers, nonlinear control components
^38^	Analytical	Insertion loss	Flexible	Combination	Wide	NR	Transformers used, fully integrated, nonlinear control components, complex mathematical modeling
This work	Numerical	Insertion loss	Flexible	Combination	Wide	4.9 µs	Automated tuning, no need of complex mathematical modeling, linear control components, digital circuitry, simple analogue components, reliable convergence, reduction in insertion-loss, autonomous control of adaptive antenna matching networks, optimum power transfer, fully integrated

Combination: passive and active; NR: not reported.

## Conclusion

Adaptive impedance matching technique is proposed that controls reactive elements in an *LC*-network for automatic compensation of fluctuations in antenna impedance. By cascading the two control loops we can achieve independent control of the real and the imaginary-parts of the antenna impedance for fast convergence. An algorithm was written to verify the effectiveness of the technique with a wide range of antenna loads. In the simulation appropriate range of the capacitance values were used for the varactor diodes. Convergence to the required target antenna load impedance was reached within 5 µs and the mismatch error with was less than 0.2%. Prior to integration of the proposed technique in mobile wireless systems consideration will need to be given on how the impedance matching improvement is offset by loss introduced by its implementation.
